# Network epidemiology and plant trade networks

**DOI:** 10.1093/aobpla/plu007

**Published:** 2014-02-18

**Authors:** Marco Pautasso, Mike J. Jeger

**Affiliations:** 1 Forest Pathology and Dendrology, Institute of Integrative Biology, ETHZ, Zurich, Switzerland; 2 Division of Ecology and Evolution & Centre for Environmental Policy, Imperial College London, London, UK

**Keywords:** Complex networks, epidemic threshold, global change, *Hymenoscyphus pseudoalbidus*, infectious diseases, information diffusion, network structure, *Phytophthora ramorum*, scale-free, small-world.

## Abstract

Models of epidemics in complex networks are improving our predictive understanding of infectious disease outbreaks. Nonetheless, applying network theory to plant pathology is still a challenge. This overview summarizes some key developments in network epidemiology that are likely to facilitate its application in the study and management of plant diseases. Recent surveys have provided much-needed datasets on contact patterns and human mobility in social networks, but plant trade networks are still understudied. Human (and plant) mobility levels across the planet are unprecedented—there is thus much potential in the use of network theory by plant health authorities and researchers. Given the directed and hierarchical nature of plant trade networks, there is a need for plant epidemiologists to further develop models based on undirected and homogeneous networks. More realistic plant health scenarios would also be obtained by developing epidemic models in dynamic, rather than static, networks. For plant diseases spread by the horticultural and ornamental trade, there is the challenge of developing spatio-temporal epidemic simulations integrating network data. The use of network theory in plant epidemiology is a promising avenue and could contribute to anticipating and preventing plant health emergencies such as European ash dieback.

## Abstract (precis)

Epidemic models in complex networks are helping us better understand infectious disease outbreaks. This review focuses on the application of new developments in network epidemiology to the study and management of plant diseases. The main aspects covered are: 1) surveys of social networks, 2) models and data about human mobility, 3) epidemic models in directed and hierarchical networks, 4) studies of dynamic networks, and 5) spatial epidemic simulations integrating network data. Because of the increasing amounts of traded plant commodities and the associated rise in introduced plant pests and pathogens, network theory has a great potential in plant science.

## Introduction

**Figure 1.**
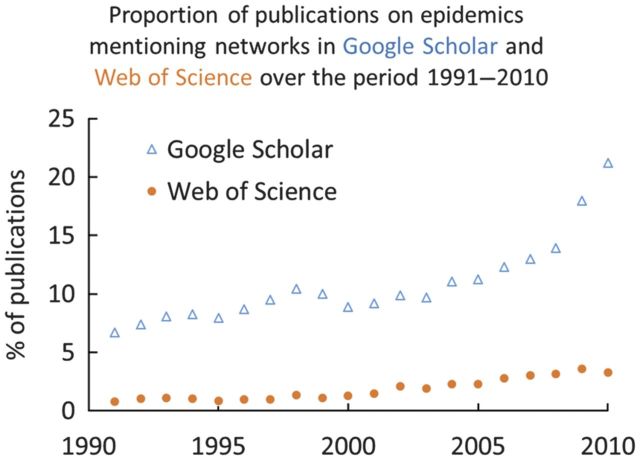
Temporal trend in the proportion of epidemiological publications mentioning networks (obtained by dividing the number of papers retrieved each year searching at the same time for the keywords ‘networks’ and ‘epidemic’ by the number of papers retrieved that year with the keyword ‘epidemic’), in Google Scholar and Web of Science (1991–2010, as abstracts are searched in Web of Science starting from 1991 only; some papers published after 2010 may still need to be indexed).

Networks are sets of nodes connected by links (Table 1). They provide a flexible framework to study emerging properties of systems composed of discrete units in the biological (Jacobi and Jonsson 2011; Latty *et al.* 2011), medical (Grassly and Fraser 2008; Gleeson *et al.* 2012), social (Newman and Park 2003; Derzsi *et al.* 2011) and technological sciences (Schweitzer *et al.* 2009; Vespignani 2012). Networks are increasingly used as models to study the spread of infectious diseases through host populations (reviewed in Kretzschmar 2000; Newman 2002; Keeling 2005; Danon *et al.* 2011; House and Keeling 2011; Robinson *et al.* 2012; Fig. 1), thus adding realism to models previously assuming that the susceptible individuals of a population were equally likely to be in contact with infectious individuals.

**Table 1.** A selection of key terms in network epidemiology (see Newman 2003 and the references in the table for further definitions and details).

Term	Explanation	Example of reference(s)
Adjacency matrix	A table summarizing the presence or absence of a link between nodes in a network	Hummon and Doreian (1990)
Basic reproduction number (*R*_0_)	The average number of individuals infected by an infectious individual in a completely susceptible population. Typically, *R*_0_ = 1 is the epidemic threshold in a homogeneous population, but this result is not valid in the presence of heterogeneity in the network contact structure	Heesterbeek (2002), May (2006)
Clustering of a network	The extent to which nodes connected to any node *x* are connected to each other	Boccaletti *et al.* (2006)
Connectance	The number of links present in a network divided by the maximum potential number of links in that network (i.e. the squared number of nodes)	Borrett and Patten (2003)
Contact network	The network of interactions among individuals. A contact is a realized link. Contact structures are equivalent to network structures	Rohani *et al.* (2010)
Degree distribution	Frequency distribution of the number of links per node. In scale-free networks, the degree distribution is well described by a power law (due to the presence of super-connected nodes). Power-law degree distributions can be caused by preferential attachment mechanisms (new nodes becoming connected to nodes that already have more links than others)	May (2006)
Epidemic threshold	The boundary (e.g. in the number of contacts or the probability of infection transmission) between no epidemic and epidemic development	Brès (1986)
Meta-population	A network of connected populations, rather than of individuals	Hess (1996)
Modularity	The degree of correlation between the probability of having a link joining nodes *x* and *y* and whether nodes *x* and *y* belong to the same community	Newman (2006)
Network	A set of individuals connected by links. If the links are asymmetric, the network is directed	Danon *et al.* (2011)
Shortest path length	The shortest number of steps (links between nodes) needed to move from node *x* to node *y*	Frank (1969)
Transmission trees	A reconstruction of the transmission events between hosts	Ypma *et al.* (2013)

One of the main results of network epidemiological studies is that network structure can have a strong influence on epidemic dynamics (Morris and Kretzschmar 1995; Christley and French 2003; Shirley and Rushton 2005; Bansal *et al.* 2007; Drewe *et al.* 2011; Smilkov and Kocarev 2012). Depending on the way links connect nodes, various network types can be distinguished (e.g. local, small-world, random and scale-free). Local networks show neighbouring connectivity, small-world networks add some rewiring of local links into long-distance connections, random networks have nodes connected with a certain probability, and scale-free networks are characterized by the presence of hubs (super-connected nodes) (Watts and Strogatz 1998; Keeling and Eames 2005; May 2006; House 2012). The presence of hubs, other things being equal, drastically reduces the epidemic threshold compared with non-scale-free network structures.

Network analyses have delivered various key insights to improve our understanding and management of epidemics (Table 2). There is a growing proportion of publications on epidemics using network approaches (Fig. 1). There is thus the need for an update, an overview of the main achievements and a summary of outstanding challenges. The aims of the present contribution are therefore to (i) selectively review key recent studies in network epidemiology, (ii) provide an overview of the main issues in this research area, and (iii) identify some major challenges for further progress in the field of plant epidemiology. The review does not aim to be comprehensive, as it attempts to draw lessons for plant epidemiology from the body of literature on diseases spreading through human networks. There is much less material on diseases spreading in networks for horticulture, grassland and forestry compared with human medicine, despite the potential of the approach in the plant sciences (Jeger *et al.* 2007; Moslonka-Lefebvre *et al.* 2011; Garrett 2012). Network applications are relatively underused in animal epidemiology too (Woolhouse *et al.* 2005; Craft and Caillaud 2011; Dubé *et al.* 2011), but less so than for plant diseases.

**Table 2.** A selection of key network theory insights about disease management.

Insight	Explanation	Reference
No epidemic threshold in scale-free networks of infinite size	In infinite-size networks with super-connected nodes, pathogens are able to spread no matter how low their infectivity is, because the low transmission rate is counteracted by the efficiency of the scale-free structure	Pastor-Satorras and Vespignani (2001)
Resistance and vulnerability of scale-free networks	Disease spread in scale-free networks is generally resistant to random removal of nodes, but vulnerable to targeted control of hubs	May (2006)
Acquaintance immunization	Super-connected nodes can be identified by randomly choosing individuals and tracing their contacts because, by definition, hubs have more connections than other nodes	Cohen *et al.* (2003)
Network topology↔epidemic spread	Epidemics are affected by network structure, but also tend to modify the patterns of contacts from infected to susceptible individuals	Kamp (2010*a*)
Host behavioural changes can amplify epidemic cycles	If mild epidemics are followed the next year by low vaccination rates, and vice versa for severe epidemics, there is the potential for an amplification of natural epidemic cycles	Cornforth *et al.* (2011)
Vaccination makes sense if connectivity is local	For an outbreak to be contained by voluntary vaccination only, infection transmission has to happen through close contacts only	Perisic and Bauch (2009)
Epidemics are more likely in a small-world network	Heterogeneity in commuting distances between individuals reduces the critical threshold for epidemics	Balcan and Vespignani (2012)
A static network structure can sometimes be realistic	If demographic and social changes do not result in substantial changes to the network (at least compared with the rate of spread of the disease), then a fixed network structure can be a realistic first approximation	Christensen *et al.* (2010)
The identity of moving individuals matters	Meta-population epidemic models that keep track of the identity of moving individuals result in spatial spread of disease reduced by ∼20 %	Keeling *et al.* (2010)
Lower epidemic threshold in dynamic small-world networks	Small-world networks with dynamic long-distance links show a higher probability of disease spread (equivalent to adding 20 % more short-cuts in a static small-world network)	Ochab and Gora (2011)
Real-world small-world networks are also slow	Community structure and the temporal dynamics of links slow down the spread of diseases in real-world small-world networks	Karsai *et al.* (2011)

The review discusses five major types of challenges relevant to

- defining contact networks related to epidemics,
- assessing the consequences for diseases of modern mobility,
- studying disease spread and control in directed, hierarchical networks,
- moving from static to dynamic network models, and
- developing spatio-temporal network simulations of diseases.

The review concludes by pointing out challenges due to the interdisciplinarity of the field and the need to ensure long-term funding for research and review in the network epidemiology of plant diseases.

## Contact Patterns

Network structures are a key factor in epidemics (Eames and Keeling 2002; Christensen *et al.* 2010). Much effort is thus being invested to improve our understanding of real-world contacts between human beings (Ames *et al.* 2011; Potter *et al.* 2011; Oliveira and Gama 2012). Contacts are interactions between individuals which create a network (Table 1). Methods used to gather these data and key challenges in their application in modelling infectious diseases have been reviewed by Barrat *et al.* (2014).

For example, a diary-based survey of close interactions of a sample of the human population in different European countries showed a high degree of age assortativity, i.e. the tendency of individuals of a given age range to interact with other individuals of the same age range (Mossong *et al.* 2008). Not taking into account age assortativity in epidemic models of pertussis has been shown to lead to misleading policy recommendations (Rohani *et al.* 2010). High-resolution contact patterns were recently obtained from a French school using sensing technology over 2 days (with an average of 323 contacts per day per participant; Stehlé *et al.* 2011) and from an Italian paediatrics hospital ward during a 1-week period (with a median of 20 contacts per day per participant; Isella *et al.* 2011).

Heterogeneities in the number of links are fundamental not just within a population, but also among different population subgroups (Conlan *et al.* 2011). Also the duration of a contact matters, for example because an infected individual will not be able to infect a new individual if it keeps staying in contact with those that have already been infected (Miller *et al.* 2012). A postal and online survey of the contacts of 5000 citizens in England, Scotland and Wales found high levels of clustering (the extent to which contacts are transitive; Table 1), which increased with the frequency and duration of contacts (Danon *et al.* 2013). Data on the frequency and duration of individual-to-individual interactions were recorded at scientific conferences, with a tendency for most interactions to be ephemeral (Zhao *et al.* 2011). In the European diary-based survey, the majority of daily contacts lasted an hour or more, but this length was reduced to <15 min for the majority of contacts with previously unknown people (Mossong *et al.* 2008). This implies that contacts tend to be longer within clusters of human individuals than across them.

It is now accepted that the number of different contacts (the degree of a node) is one of the key indicators of the risk to acquire and further spread a disease (Salathé *et al.* 2010), but other network metrics, e.g. repeated exposure, can be just as important (Iozzi *et al.* 2010). A major challenge remains in inferring network properties from sampling data obtained from a limited part of contact networks (Stumpf *et al.* 2005; Annibale and Coolen 2011; Nickbakhsh *et al.* 2011). Variation in sampling effort among studies mapping networks is a source of uncertainty also outside epidemiology, e.g. in anthropology, food web ecology and geography (Dormann *et al.* 2009). Different types of contacts (e.g. family, healthcare, sexual, social, travel, virtual contacts) will tend to be associated with different probabilities of disease transmission across these contacts for different types of disease (Rothenberg and Narramore 1996; Smith and Christakis 2008; Smieszek 2010; Bolton *et al.* 2012).

Different types of contacts are found in plant trade networks too, if only because of the diversity of traded plant species and types (cut flowers, plants for planting, bare-rooted saplings, ornamental twigs, fruits, etc.) (Dehnen-Schmutz *et al.* 2010). Inferring the properties of a whole network when only sampling parts of it is an issue for plant trade networks—just as for human social networks—because of the arbitrariness of social and trade network boundaries, and also due to the general lack of standardized data on plant trade networks. It is likely that contacts among plant traders (e.g. nurseries, garden centres and supermarkets) are more long-lasting than contacts among researchers at a conference, but collaboration of researchers with plant health authorities and traders is needed to prove that this is so.

Plant epidemiologists can only aspire to the data currently gathered on human contact patterns. For reasons of commercial sensitivity, plant trade networks are indeed still poorly characterized. This lack of knowledge on the structure of horticultural and ornamental trade networks makes it difficult to prevent the inadvertent dispersal of plant pathogens and pests together with the traded plant material (Helfer 2014; Schoebel *et al.* 2014). Regional and international surveys are needed to achieve reconstructions of the networks of plant shipments among plant nurseries and their customers, as well as the networks of informal plant and seed exchanges among farmers, gardeners or urban dwellers.

To assess how likely each commodity is to spread various plant diseases, we then need to overlap the trade network with interception data, which are already recorded (although not always made publicly available) at international borders. Very helpful from a plant health risk assessment perspective would be the systematic inspection of intercepted consignments, e.g. at EU borders, so as to gather data on the proportion of plant material infested by a particular pest or pathogen. But these would be costly measures and border inspectors are already overwhelmed by the increasing amounts of traded plant material (van der Graaf and Khoury 2010).

Of concern is also the lack of studies on the networks of plants and plant commodities traded on the internet (Giltrap *et al.* 2009; Pautasso *et al.* 2012; Shirey *et al.* 2013). We need to map the presence of heterogeneities in contact numbers among internet plant traders, so as to establish whether super-connected nodes are present and, in that case, targeting control at them, because this approach has been shown, for livestock diseases and vertebrate invasive species, to be more efficient and effective than other control strategies (Natale *et al.* 2009; Florance *et al.* 2011).

## Human (and Plant) Mobility

Current human mobility is massive, unprecedented, still increasing and has important consequences for the potential and actual spread of diseases over multiple scales. Not only people, but a range of associated goods, ideas and organisms are moving around the planet along modern transportation and communication networks (see reviews in Brockmann 2010; Kaluza *et al.* 2010; Woolley-Meza *et al.* 2011; Fisher *et al.* 2012).

One of the challenges of including human mobility data in epidemic models is given by the multiple types of human movements. Although regional commuting flows (e.g. train, car, bus and cycling journeys) do not make the planet a small-world (i.e. a network with a low average number of steps between two randomly chosen individuals) on their own, they are about one order of magnitude larger than airline flows. Thus, taking into account regional commuters in epidemic models leads to increased synchronization of epidemics in different parts of the planet (Balcan *et al.* 2009). This synchronization may in turn decrease opportunities for health officers, policy makers and the public in regions where a pathogen is still unreported to learn from epidemic developments in regions first hit by that pathogen.

Human mobility data are being obtained from various sources, from consecutive sightings of banknotes to mobile phone records. While banknote sightings have been useful in establishing the predictability of movement patterns on broader scales, mobile phone trajectories operating in space–time provide detailed insights on individual mobility patterns (González *et al.* 2008).

A key implication of current human mobility patterns is that epidemics of infectious diseases will spread more rapidly than in previous centuries or decades, other things being equal (Merler and Ajelli 2010*a*). However, for many human diseases other things are not equal: hygiene levels have steadily improved, and people's awareness of infectious diseases, their causes and what can be done to prevent and cure them is not what it was in the past (Smith 2007; Ashenburg 2008; Armelagos 2009). At the same time, there are factors other than globalization facilitating the emergence of new diseases. For example, many more people inhabit the planet (and its urbanized areas) than at any time in the past, antibiotic resistance has arisen repeatedly and the climate has started to warm (McMichael and Beaglehole 2000; May 2007; Jones *et al.* 2008; Harper and Armelagos 2010; Conraths and Mettenleiter 2011).

Each of these developments has consequences for epidemic development: for example, many pathogens tend to reach urban areas earlier than rural ones (Weiss and McMichael 2004; Woolhouse and Gaunt 2007; Pongsiri *et al.* 2009; Merler and Ajelli 2010*b*), so that a more urbanized planet will tend to have less time to respond to new epidemics of human diseases. A counteracting factor here is that care tends to be more accessible in urbanized regions compared with rural areas (Rabinowitz *et al.* 1999). A key research issue is how the heterogeneity of mobility (and disease management) networks can be influenced in a timely (and long-term) manner to reduce the risk posed by epidemics (Miller *et al.* 2009; Draief and Ganesh 2011).

Increasing mobility is a key issue in the network epidemiology of plant diseases, because ideally information about how to handle a new invasive plant pathogen needs to reach farmers and plant health authorities before the pathogen is introduced (Rebaudo and Dangles 2011; Garrett 2012). Often this is not the case. For example, the concurrent emergence of the epidemic of *Phytophthora ramorum* in the west coast of the USA and in Europe took place when both regions lacked epidemiological knowledge on this aggressive, generalist and new plant pathogen (Brasier and Webber 2010; Pautasso 2013; Redlin *et al.* 2014).

Plant passporting schemes and their associated trace-forward and trace-back data hold much potential to reconstruct plant mobility patterns, but this potential still waits to be exploited. Also in the case of plant health, molecular advances and international regulations are developing tools to counterbalance the risks posed by globalization, but there is a need to improve the uptake of network approaches by plant health authorities (Brasier 2008; MacLeod *et al.* 2010; Jeger *et al.* 2012; Hantula *et al.* 2014). Research is needed to test whether there is heterogeneity in the average commuting distances of plants-for-planting of different species and in various regions. A potentially confounding factor in such studies is the variation among rural and urbanized regions (as well as among industrialized and developing countries) in resources to track and fight plant epidemics. Even if new plant pathogens tend to be recorded first in urbanized regions (Yemshanov *et al.* 2013), the bulk of the cultivated area is located in rural areas, so that there might be a longer lag between first report and epidemic, thus providing some more time for rural economies to prepare for new plant health risks.

Taken together, these changes in the global health landscape imply that there is a need for further integration of human and plant mobility data in plant disease epidemic models. The growing complexity of mobility data can potentially enable more detailed epidemiological models, but these may also be more difficult to interpret and generalize, so that a compromise between realism and parsimoniousness is needed.

## Directed and Hierarchical Networks

One key assumption that is often taken in models of epidemic spread in networks is the symmetrical nature of contacts (Bianconi *et al.* 2008). This greatly simplifies simulations of epidemics in networks, but may or may not be appropriate depending on the system (reviewed in Newman *et al.* 2001; Meyers *et al.* 2006). For example, while disease transmission on transportation networks is often asymmetric, airborne infection can be thought of as symmetric without loss of generality (although asymmetry can be present for airborne dispersal depending on the prevailing wind direction and speed).

Even in the case of symmetric contact patterns (i.e. where the probability of transmission is equivalent in both directions), transmission trees (the routes followed by infection) are directed (Welch *et al.* 2011). Pathogen transmission tends to occur from one node to another, e.g. from the country of first detection to elsewhere in the world (although subsequent re-infection from elsewhere in the world to the country of first detection can well take place). For example, as a likely consequence of its special position as a transatlantic bridge between the USA and continental Europe, the UK was the only European country to experience a summer peak of the H1N1 flu in 2009 (Merler *et al.* 2011). Epidemic transmission trees are in many cases unknown, but can be reconstructed from phylogenetic information on the pathogen at various locations in the putative contact network (Mascheretti *et al.* 2009; Leventhal *et al.* 2012; Xhaard *et al.* 2012; Ypma *et al.* 2012).

The directedness of networks results in hierarchical systems where nodes can be divided into main classes. For example, nodes can have (i) more outgoing than incoming connections, (ii) more incoming than outgoing connections, or (iii) a balance between the two (Pautasso *et al.* 2010*c*). Networks relevant to disease spread which show hierarchical features in their structure include the world trade web (Ruzzenenti *et al.* 2010; Barigozzi *et al.* 2011; De Benedictis and Tajoli 2011) and subsets of it such as the horticultural and ornamental plant trade networks (Dehnen-Schmutz *et al.* 2010; Bradley *et al.* 2012; Liebhold *et al.* 2012). Food webs of producers, consumers and parasites are also inherently asymmetric and hierarchical (Foster *et al.* 2010), and so are many networks diffusing health information from researchers to physicians, policy makers and patients (Wang and Noe 2010; Zappa 2011).

In directed networks of small size (hundreds of nodes), the presence of super-connected nodes only lowers the epidemic threshold if these nodes have a high number of both incoming and outgoing links (Moslonka-Lefebvre *et al.* 2009). An open question is how small the size of networks can be for significant differences in the epidemic threshold among network structures to be present (for a given number of network replicates). In hierarchical, directed networks with node status (infectious vs. susceptible) along a continuum, the epidemic final size correlates well with the number of outgoing links of the starting node of the epidemic, regardless of network structure (local, random, small-world and scale-free) and connectance level (Pautasso *et al.* 2010*b*). Since we do not know which network structure applies to plant trade networks, the out-degree of a node can thus be a useful indicator of its risk.

A continuum between infectivity and susceptibility makes sense when nodes are composed of multiple units (e.g. pupils in school classes, patients in hospital wards, plants in plant nurseries), but such an approach has been rarely used in network epidemiology (Moslonka-Lefebvre *et al.* 2012). There is thus a challenge in inferring whether results obtained from models assuming that nodes are binary, i.e. either entirely susceptible or entirely infectious, can be transferred to plant trade networks, as only a proportion of the plants in nurseries and garden centres are typically infected. The same problem applies in plant pathology when using results obtained from epidemic simulations in symmetric networks, given that networks of plant movements are inherently directed. When plants (and their associated organisms) are moved from location *x* to location *y*, the reverse movement from location *y* to location *x* does not usually occur (Brenn *et al.* 2008). Information is needed about the frequency and dynamics, in various countries, of subgroups such as plant producers, wholesalers, retailers and end users, because their relative proportion has been shown to be related to the likelihood that an epidemic will spread (Pautasso *et al.* 2010*c*).

## Dynamic Networks

The influence of network topology on epidemic spread is not mono-directional, because, as the epidemic unfolds and individuals become infected or recover from infection, the patterns of contacts from infected to susceptible individuals will be modified (Kamp 2010*a*, *b*; House 2012). In addition, dynamic networks can result from the mobility of hosts (Song *et al.* 2010*a*; Gargiulo *et al.* 2012) and active behavioural changes in response to an epidemic (Saadatian-Elahi *et al.* 2010; Ridenhour *et al.* 2011). A review focusing on this area is provided by Funk *et al.* (2010).

**Figure 2.**
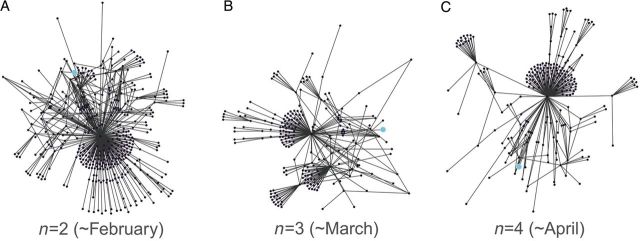
Temporal fluctuations in the contacts of an animal farm in Italy (neighbourhoods at distance = 3 of the same node (shown in light blue) in three consecutive monthly networks; modified from Bajardi *et al.* (2011*a*), with kind permission of the Public Library of Science). The assessment of the epidemiological consequences of such network dynamics is a challenge for human, animal and plant diseases.

Despite the many sources of network dynamics, most epidemiological models have assumed so far that the structure of a network is fixed (Grindrod and Higham 2010). A similar assumption is that demographic processes (e.g. birth, migration, marriage, death) do not result in substantial changes in the network (at least compared with the rate of spread of the disease; Christensen *et al.* 2010). However, taking into account the dynamic nature of social networks can lead to different model outcomes or data requirements (Fefferman and Ng 2007; Vernon and Keeling 2009; Volz and Meyers 2009; Fig. 2).

In small-world networks, dynamically rewiring long-distance links during an epidemic leads to a higher probability of disease spread (equivalent to adding 20 % more short-cuts; Ochab and Gora 2011). Incidentally, a dynamic small-world network is computationally more tractable than a fixed one, because adjacency matrices are no longer required; all that is needed is the probability for a short-cut to be present (Stone and McKay 2011). This can be a disadvantage though, if the aim is to model adaptive rewiring (the transformation of local connections into long-distance ones in an adaptive way) in response to an epidemic, because with that approach the long-distance connections are chosen randomly (Stone *et al.* 2010).

Some epidemic models taking into account human mobility are dynamically quenched, which means that if an individual visits a new location, the same individual will be more likely than not to return to that place in the future (Song *et al.* 2010*b*). Even if this is the case, the topological distance (the shortest path separating nodes; Table 1) may differ in static vs. temporal networks. Temporal networks are those taking into account changes in the contacts through time. For example, an alternative way to create a dynamic (small-world) network is to simulate disease spread on 365 static (but different) networks, one for each day of the year (Vernon and Keeling 2009). Nodes that appear to be central in static networks may be of limited importance in spreading disease when the temporal fluctuations in contact patterns are considered (Pan and Saramäki 2011). Whether this result also applies to plant trade networks is to the best of our knowledge still to be tested.

For both human and plant diseases, a challenge in modelling disease spread in dynamic networks is the use of metrics that can account for dynamic properties (Natale *et al.* 2011), given that most network metrics are based on static networks (Bajardi *et al.* 2011*b*; Nöremark *et al.* 2011; Klemm *et al.* 2012). In addition, it would be interesting to know whether temporal preferential attachment mechanisms (the longer a node is connected to another node, the more the two nodes are likely to be connected in the future; Stehlé *et al.* 2010) apply also to plant traders.

Nowadays, traded plants and plant parts are no less mobile than human beings, and changes in the behaviour of the industry in response to plant epidemics are observed also in the trade of plants (e.g. switching from susceptible to resistant plant hosts). Research on how long-distance plant trade connections emerge in practice and whether they tend to be maintained through time would be needed. For example, dynamic network models of the epidemic of soybean rust (*Phakopsora pachyrhizi*) in the USA, with counties as nodes and links weighted in proportion to host cultivated area and wind speed and direction, resulted in a reduction of the resources needed to track and predict the crop epidemic (Sutrave *et al.* 2012). This is a good example of how network models can be used without an underlying trade network.

## Spatio-temporal Network Simulations

For disease control to be informed and to benefit from epidemic simulations in networks, these need to be grounded in realistic spatial and temporal settings (Viboud *et al.* 2006; Harwood *et al.* 2009; Dent *et al.* 2011). A review of the effects of spatial structure on infectious disease dynamics is provided by Rock *et al.* (2014).

Networks have traditionally been treated as abstract constructs, given their origin in mathematical topology (Dale and Fortin 2010; Rozhnova *et al.* 2011). However, where a group of contacts is located and how it evolves in time can make epidemic outcomes differ from what would have been predicted at an abstract network level (Bertuzzo *et al.* 2010). Similarly, superimposing contact networks on spatial epidemic models can improve overall predictions. For example, the peak in influenza-like incidence reported in Italy in 2009 was shown to have occurred earlier in regions with a higher number of arriving international air-bound passengers (Ajelli *et al.* 2011).

The key role of space does not just apply to epidemics in networks, because many biological and social networks are also spatially embedded (Barthélemy 2011; Expert *et al.* 2011). However, for epidemics, more so than for other systems, there is often the necessity to assess in real time (while the epidemic is ongoing) the potential impact over geographical regions (Birrell *et al.* 2011). This is difficult also for human diseases given the frequent lack of good data at the beginning of an epidemic (Katriel *et al.* 2011). Network approaches can help trace back the origin of epidemics. For example, an airline network perspective makes it possible to recover an expanding wave of infection around the airport from which an epidemic started spreading, whereas that origin would not be easily traceable when following the same epidemic on a spatial map of the world (Brockmann and Helbing 2013).

A step forward would be the development of software for contemporaneous temporal comparison of different policy measures (Maciejewski *et al.* 2011). There is the need to test the effectiveness of the use of such visual tools by researchers and public health officials in the evaluation of measures to respond to epidemic outbreaks (Afzal *et al.* 2011). Together with better data at the early epidemic stages, spatio-temporal simulations are needed to make progress in assessing how behavioural changes and the spread of health information affect the resilience of networks (Birrell *et al.* 2011; Döring *et al.* 2014). Also control measures tend to differ in space and time, so that an important issue to be explored is the interplay of dynamic disease control measures in network epidemic models (Green *et al.* 2011).

Epidemic simulations and adaptive disease management modelling in real time are still unrealistic for plant epidemics occurring in trade networks for which there is no information about the network structure, particularly when disease monitoring is patchy and reporting is slower than the epidemic. However, molecular advances (e.g. real-time PCR) and citizen-science approaches (e.g. in the mapping of emerging diseases across whole countries) could make real-time simulations possible for plant epidemics too. A spatio-temporal approach is already inherent in empirical analyses of plant pathogens introduced by trade networks into the wider environment. For example, a study of first records of plant pathogens in the UK (1970–2004) showed that the Netherlands was the origin of introductions (when known) nearly half of the time, and the South-East of England was the region of first report (when known) for 38 % of newly recorded pathogens (Jones and Baker 2007). For historical and geographical reasons, the Netherlands and the South-East of England have more trade connections than other European countries and British regions, respectively.

This kind of analysis can provide useful data to parameterize spatial epidemic network models. Similarly, a temporal study of the spatial association in England and Wales (2003–06) between *P. ramorum* reports in the trade and in the wild found that infected premises (garden centres and nurseries) were significantly associated with infected sites (woodlands and historic gardens) at short distances (<1 km) over the studied period, thus suggesting a role of the trade in spreading the epidemic (Xu *et al.* 2009). Given that various *Phytophthora* species are found in plant nurseries, and that some of these species have hybridized to produce new emerging pathogens, plant epidemiology too may benefit from models of the interactions between different pathogen strains spreading in spatial networks (Alexander and Kobes 2011; Dorigatti and Pugliese 2011; Karrer and Newman 2011; Weng *et al.* 2012).

## Conclusions

A major issue for spatially explicit modelling of diseases spreading in networks is the communication of its inherent uncertainty and simplifications to end users (policy makers, health providers, journalists, patients) (Prieto *et al.* 2012). Despite the growing sophistications, spatial models of epidemics in networks are still simplifications of a complicated system (Reppas *et al.* 2010). Only rarely is this acknowledged in the discussion of papers reporting results from models of epidemics in spatial networks, so that there is a danger of take-home messages being read without keeping in mind all the various caveats and assumptions of the mathematical models behind policy recommendations (Ferguson *et al.* 2006; Fraser *et al.* 2009; Garnett *et al.* 2011).

Nevertheless, networks provide a flexible framework that can link disciplines and practitioners working in different domains, for example human and plant pathologists, as well as epidemiologists and invasion biologists (Borer *et al.* 2011; Kueffer *et al.* 2013). Network theory is a useful addition to the set of tools available to understand and manage (plant) diseases, particularly when pathogens are dispersed by human movements over long distances (Jeger *et al.* 2007). This review has focused on the use of networks to model the plant trade, but network theory in plant epidemiology can also be applied at the genetic, molecular, meta-population and landscape levels (Moslonka-Lefebvre *et al.* 2011).

One important research area where network theory is likely to prove fruitful is the integration of plant health models predicting climate change impacts with those assessing the likely effects of globalization on emerging plant diseases (Pautasso *et al.* 2010*a*). Long-term funding for plant health modellers, experimenters and practitioners would be needed to develop this research programme. Nevertheless, an improved understanding of the structure and dynamics of plant trade networks can already provide useful insights before the expected climate changes of the coming decades take place. For example, the recent establishment of European ash dieback (caused by *Hymenoscyphus pseudoalbidus*; Queloz *et al.* 2011) throughout Europe was facilitated by local and long-distance trade in infected ash saplings, which continued to be planted in forests and plantations for several years despite the growing evidence that tree nurseries were a pathway for the spread of this emerging disease (Gross *et al.* 2014).

## Sources of Funding

Our work was partly funded by the Rural Economy and Land Use (RELU) programme and the French Foundation for Research on Biodiversity (FRB) through its Centre for Analysis and Synthesis of Biodiversity Data (CESAB).

## Contributions by the Authors

All authors have made a substantial contribution to the manuscript. Each author has seen and agreed to the submitted manuscript.

## Conflicts of Interest Statement

None declared.

## References

[PLU007C1] Afzal S, Maciejewski R, Ebert DS (2011). Visual analytics decision support environment for epidemic modeling and response evaluation. *2011 IEEE Conference on Visual Analytics Science and Technology (VAST)*.

[PLU007C2] Ajelli M, Merler S, Pugliese A, Rizzo C (2011). Model predictions and evaluation of possible control strategies for the 2009 A/H1N1v influenza pandemic in Italy. Epidemiology & Infection.

[PLU007C3] Alexander ME, Kobes R (2011). Effects of vaccination and population structure on influenza epidemic spread in the presence of two circulating strains. BMC Public Health.

[PLU007C4] Ames GM, George DB, Hampson CP, Kanarek AR, McBee CD, Lockwood DR, Achter JD, Webb CT (2011). Using network properties to predict disease dynamics on human contact networks. Proceedings of the Royal Society B Biological Sciences.

[PLU007C5] Annibale A, Coolen ACC (2011). What you see is not what you get: how sampling affects macroscopic features of biological networks. Interface Focus.

[PLU007C6] Armelagos GJ, Rook GAW (2009). The Paleolithic disease-scape, the hygiene hypothesis, and the second epidemiological transition. The hygiene hypothesis and Darwinian medicine.

[PLU007C7] Ashenburg K (2008). Clean: an unsanitized history of washing.

[PLU007C8] Bajardi P, Barrat A, Natale F, Savini L, Colizza V (2011a). Dynamical patterns of cattle trade movements. PLoS One.

[PLU007C9] Bajardi P, Poletto C, Ramasco JJ, Tizzoni M, Colizza V, Vespignani A (2011b). Human mobility networks, travel restrictions, and the global spread of 2009 H1N1 pandemic. PLoS One.

[PLU007C10] Balcan D, Vespignani A (2012). Invasion threshold in structured populations with recurrent mobility patterns. Journal of Theoretical Biology.

[PLU007C11] Balcan D, Colizza V, Gonçalves B, Hu H, Ramasco JJ, Vespignani A (2009). Multiscale mobility networks and the spatial spreading of infectious diseases. Proceedings of the National Academy of Sciences of the USA.

[PLU007C12] Bansal S, Grenfell BT, Meyers LA (2007). When individual behaviour matters: homogeneous and network models in epidemiology. Interface.

[PLU007C13] Barigozzi M, Fagiolo G, Mangioni G (2011). Identifying the community structure of the international-trade multi-network. Physica A.

[PLU007C14] Barrat A, Cattuto C, Tozzi AE, Vanhems P, Voirin N (2014). Measuring contact patterns with wearable sensors: methods, data characteristics and applications to data-driven simulations of infectious diseases. Clinical Microbiology and Infection.

[PLU007C15] Barthélemy M (2011). Spatial networks. Physics Reports.

[PLU007C16] Bertuzzo E, Casagrandi R, Gatto M, Rodriguez-Iturbe I, Rinaldo A (2010). On spatially explicit models of cholera epidemics. Interface.

[PLU007C17] Bianconi G, Gulbahce N, Motter AE (2008). Local structure of directed networks. Physical Review Letters.

[PLU007C18] Birrell PJ, Ketsetzis G, Gay NJ, Cooper BS, Presanis AM, Harris RJ, Charlett A, Zhang X-A, White PJ, Pebody RG, De Angelis D (2011). Bayesian modeling to unmask and predict influenza A/H1N1pdm dynamics in London. Proceedings of the National Academy of Sciences of the USA.

[PLU007C19] Boccaletti S, Latora V, Moreno Y, Chavez M, Hwang DU (2006). Complex networks: structure and dynamics. Physics Reports.

[PLU007C20] Bolton KJ, Mccaw JM, Forbes K, Nathan P, Robins G, Pattison P, Nolan T, Mcvernon J (2012). Influence of contact definitions in assessment of the relative importance of social settings in disease transmission risk. PLoS One.

[PLU007C21] Borer ET, Antonovics J, Kinkel LL, Hudson PJ, Daszak P, Ferrari MJ, Garrett KA, Parrish CR, Read AF, Rizzo DM (2011). Bridging taxonomic and disciplinary divides in infectious disease. Ecohealth.

[PLU007C22] Borrett SR, Patten BC (2003). Structure of pathways in ecological networks: relationships between length and number. Ecological Modelling.

[PLU007C23] Bradley BA, Blumenthal DM, Early R, Grosholz ED, Lawler JJ, Miller LP, Sorte CJB, D'Antonio C, Diez JM, Dukes JS, Ibanez I, Olden JD (2012). Global change, global trade, and the next wave of plant invasions. Frontiers in Ecology and the Environment.

[PLU007C24] Brasier C, Webber J (2010). Sudden larch death. Nature.

[PLU007C25] Brasier CM (2008). The biosecurity threat to the UK and global environment from international trade in plants. Plant Pathology.

[PLU007C26] Brenn N, Menkis A, Grünig CR, Sieber TN, Holdenrieder O (2008). Community structure of *Phialocephala fortinii*s. lat. in European tree nurseries, and assessment of the potential of the seedlings as dissemination vehicles. Mycological Research.

[PLU007C27] Brès P (1986). Public health action in emergencies caused by epidemics.

[PLU007C28] Brockmann D (2010). The physics of where to go. Nature Physics.

[PLU007C29] Brockmann D, Helbing D (2013). The hidden geometry of complex, network-driven contagion phenomena. Science.

[PLU007C30] Christensen C, Albert I, Grenfell B, Albert R (2010). Disease dynamics in a dynamic social network. Physica A.

[PLU007C31] Christley RM, French NP (2003). Small-world topology of UK racing: the potential for rapid spread of infectious agents. Equine Veterinary Journal.

[PLU007C32] Cohen R, Havlin S, Ben-Avraham D (2003). Efficient immunization strategies for computer networks and populations. Physical Review Letters.

[PLU007C33] Conlan AJK, Eames KTD, Gage JA, Von Kirchbach JC, Ross JV, Saenz RA, Gog JR (2011). Measuring social networks in British primary schools through scientific engagement. Proceedings of the Royal Society B Biological Sciences.

[PLU007C34] Conraths FJ, Mettenleiter TC (2011). Infectious diseases under the influence of changing environmental factors. Progress in Parasitology.

[PLU007C35] Cornforth DM, Reluga TC, Shim E, Bauch CT, Galvani AP, Meyers LA (2011). Erratic flu vaccination emerges from short-sighted behavior in contact networks. PLoS Computational Biology.

[PLU007C36] Craft ME, Caillaud D (2011). Network models: an underutilized tool in wildlife epidemiology?. Interdisciplinary Perspectives on Infectious Diseases.

[PLU007C37] Dale MRT, Fortin MJ (2010). From graphs to spatial graphs. Annual Review of Ecology, Evolution & Systematics.

[PLU007C38] Danon L, Ford AP, House T, Jewell CP, Keeling MJ, Roberts GO, Ross JV, Vernon MC (2011). Networks and the epidemiology of infectious disease. Interdisciplinary Perspectives on Infectious Diseases.

[PLU007C39] Danon L, Read JM, House TA, Vernon MC, Keeling MJ (2013). Social encounter networks: characterizing Great Britain. Proceedings of the Royal Society B Biological Sciences.

[PLU007C40] De Benedictis L, Tajoli L (2011). The world trade network. The World Economy.

[PLU007C41] Dehnen-Schmutz K, Holdenrieder O, Jeger MJ, Pautasso M (2010). Structural change in the international horticultural industry: some implications for plant health. Scientia Horticulturae.

[PLU007C42] Dent JE, Kiss IZ, Kao RR, Arnold M (2011). The potential spread of highly pathogenic avian influenza virus via dynamic contacts between poultry premises in Great Britain. BMC Veterinary Research.

[PLU007C43] Derzsi A, Derzsy N, Káptalan E, Néda Z (2011). Topology of the Erasmus student mobility network. Physica A.

[PLU007C44] Dorigatti I, Pugliese A (2011). Analysis of a vaccine model with cross-immunity: when can two competing infectious strains coexist?. Mathematical Biosciences.

[PLU007C45] Döring TF, Vieweger A, Pautasso M, Vaarst M, Finckh MR, Wolfe MS (2014). Resilience as a universal criterion of health. Journal of the Science of Food and Agriculture.

[PLU007C46] Dormann CF, Fründ J, Blüthgen N, Gruber B (2009). Indices, graphs and null models: analyzing bipartite ecological networks. Open Ecology Journal.

[PLU007C47] Draief M, Ganesh A (2011). A random walk model for infection on graphs: spread of epidemics rumours with mobile agents. Discrete Events & Dynamical Systems.

[PLU007C48] Drewe JA, Eames KTD, Madden JR, Pearce GP (2011). Integrating contact network structure into tuberculosis epidemiology in meerkats in South Africa: implications for control. Preventive Veterinary Medicine.

[PLU007C49] Dubé C, Ribble C, Kelton D, Mcnab B (2011). Introduction to network analysis and its implications for animal disease modelling. CIE Revue Scientifique et Technique.

[PLU007C50] Eames KTD, Keeling MJ (2002). Modeling dynamic and network heterogeneities in the spread of sexually transmitted diseases. Proceedings of the National Academy of Sciences of the USA.

[PLU007C51] Expert P, Evans TS, Blondel VD, Lambiotte R (2011). Uncovering space-independent communities in spatial networks. Proceedings of the National Academy of Sciences of the USA.

[PLU007C52] Fefferman NH, Ng KL (2007). How disease models in static networks can fail to approximate disease in dynamic networks. Physical Review E.

[PLU007C53] Ferguson NM, Cummings DAT, Fraser C, Cajka JC, Cooley PC, Burke DS (2006). Strategies for mitigating an influenza pandemic. Nature.

[PLU007C54] Fisher MC, Henk DA, Briggs CJ, Brownstein JS, Madoff LC, McCraw SL, Gurr SJ (2012). Emerging fungal threats to animal, plant and ecosystem health. Nature.

[PLU007C55] Florance D, Webb JK, Dempster T, Kearney MR, Worthing A, Letnic M (2011). Excluding access to invasion hubs can contain the spread of an invasive vertebrate. Proceedings of the Royal Society B Biological Sciences.

[PLU007C56] Foster JG, Foster DV, Grassberger P, Paczuski M (2010). Edge direction and the structure of networks. Proceedings of the National Academy of Sciences of the USA.

[PLU007C57] Frank H (1969). Shortest paths in probabilistic graphs. Operations Research.

[PLU007C58] Fraser C, Donnelly CA, Cauchemez S, Hanage WP, Van Kerkhove MD, Hollingsworth TD, Griffin J, Baggaley RF, Jenkins HE, Lyons EJ, Jombart T, Hinsley WR, Grassly NC, Balloux F, Ghani AC, Ferguson NM, Rambaut A, Pybus OG, Lopez-Gatell H, Alpuche-Aranda CM, Bojorquez Chapela I, Palacios Zavala E, Espejo Guevara DM, Checchi F, Garcia E, Hugonnet S, Roth C, The WHO Rapid Pandemic Assessment Collaboration (2009). Pandemic potential of a strain of influenza A (H1N1): early findings. Science.

[PLU007C59] Funk S, Salathe M, Jansen VAA (2010). Modelling the influence of human behaviour on the spread of infectious diseases: a review. Interface.

[PLU007C60] Gargiulo F, Lenormand M, Huet S, Baqueiro Espinosa O (2012). Commuting network models: getting the essentials. Journal of Artificial Societies and Social Simulation.

[PLU007C61] Garnett GP, Cousens S, Hallett TB, Steketee R, Walker N (2011). Mathematical models in the evaluation of health programmes. Lancet.

[PLU007C62] Garrett KA (2012). Information networks for disease: commonalities in human management networks and within-host signalling networks. European Journal of Plant Pathology.

[PLU007C63] Giltrap N, Eyre D, Reed P (2009). Internet sales of plants for planting—an increasing trend and threat?. EPPO Bulletin.

[PLU007C64] Gleeson JP, Melnik S, Ward JA, Porter MA, Mucha PJ (2012). Accuracy of mean-field theory for dynamics on real-world networks. Physical Review E.

[PLU007C65] González MC, Hidalgo CA, Barabási AL (2008). Understanding individual human mobility patterns. Nature.

[PLU007C66] Grassly N, Fraser C (2008). Mathematical models of infectious disease transmission. Nature Reviews Microbiology.

[PLU007C67] Green DM, Werkman M, Munro LA, Kao RR, Kiss IZ, Danon L (2011). Tools to study trends in community structure: application to fish and livestock trading networks. Preventive Veterinary Medicine.

[PLU007C68] Grindrod P, Higham DJ (2010). Evolving graphs: dynamical models, inverse problems and propagation. Proceedings of the Royal Society A.

[PLU007C69] Gross A, Holdenrieder O, Pautasso M, Queloz V, Sieber TN (2014). *Hymenoscyphus pseudoalbidus*, the causal agent of European ash dieback. Molecular Plant Pathology.

[PLU007C70] Hantula J, Müller MM, Uusivuori J (2014). International plant trade associated risks: laissez-faire or novel solutions. Environmental Science & Policy.

[PLU007C71] Harper K, Armelagos G (2010). The changing disease-scape in the third epidemiological transition. International Journal of Environmental Research and Public Health.

[PLU007C72] Harwood TD, Xu XM, Pautasso M, Jeger MJ, Shaw M (2009). Epidemiological risk assessment using linked network and grid based modelling: *Phytophthora ramorum* and *Phytophthora kernoviae* in the UK. Ecological Modelling.

[PLU007C73] Heesterbeek JAP (2002). A brief history of R_0_ and a recipe for its calculation. Acta Biotheoretica.

[PLU007C74] Helfer S (2014). Rust fungi and global change. New Phytologist.

[PLU007C75] Hess G (1996). Disease in metapopulation models: implications for conservation. Ecology.

[PLU007C76] House T (2012). Modelling epidemics on networks. Contemporary Physics.

[PLU007C77] House T, Keeling MJ (2011). Insights from unifying modern approximations to infections on networks. Interface.

[PLU007C78] Hummon NP, Doreian P (1990). Computational methods for social network analysis. Social Networks.

[PLU007C79] Iozzi F, Trusiano F, Chinazzi M, Billari FC, Zagheni E, Merler S, Ajelli M, Del Fava E, Manfredi P (2010). Little Italy: an agent-based approach to the estimation of contact patterns-fitting predicted matrices to serological data. PLoS Computational Biology.

[PLU007C80] Isella L, Romano M, Barrat A, Cattuto C, Colizza V, Van Den Broeck W, Gesualdo F, Pandolfi E, Rava L, Rizzo C, Tozzi AE (2011). Close encounters in a pediatric ward: measuring face-to-face proximity and mixing patterns with wearable sensors. PLoS One.

[PLU007C81] Jacobi MN, Jonsson PR (2011). Optimal networks of nature reserves can be found through eigenvalue perturbation theory of the connectivity matrix. Ecological Applications.

[PLU007C82] Jeger M, Schans J, Lövei GL, van Lenteren J, Navajas M, Makowski D, Stancanelli G, Tramontini S, Ceglarska EB (2012). Risk assessment in support of plant health. EFSA Journal.

[PLU007C83] Jeger MJ, Pautasso M, Holdenrieder O, Shaw MW (2007). Modelling disease spread and control in networks: implications for plant sciences. New Phytologist.

[PLU007C84] Jones DR, Baker RHA (2007). Introductions of non-native plant pathogens into Great Britain, 1970–2004. Plant Pathology.

[PLU007C85] Jones KE, Patel NG, Levy MA, Storeygard A, Balk D, Gittleman JL, Daszak P (2008). Global trends in emerging infectious diseases. Nature.

[PLU007C86] Kaluza P, Kölzsch A, Gastner MT, Blasius B (2010). The complex network of global cargo ship movements. Interface.

[PLU007C87] Kamp C (2010a). Demographic and behavioural change during epidemics. Procedia Computer Science.

[PLU007C88] Kamp C (2010b). Untangling the interplay between epidemic spread and transmission network dynamics. PLoS Computational Biology.

[PLU007C89] Karrer B, Newman MEJ (2011). Competing epidemics on complex networks. Physical Review E.

[PLU007C90] Karsai M, Kivelä M, Pan RK, Kaski K, Kertesz J, Barabasi AL, Saramäki J (2011). Small but slow world: how network topology and burstiness slow down spreading. Physical Review E.

[PLU007C91] Katriel G, Yaari R, Huppert A, Roll U, Stone L (2011). Modelling the initial phase of an epidemic using incidence and infection network data: 2009 H1N1 pandemic in Israel as a case study. Interface.

[PLU007C92] Keeling MJ (2005). The implications of network structure for epidemic dynamics. Theoretical Population Biology.

[PLU007C93] Keeling MJ, Eames KTD (2005). Networks and epidemic models. Interface.

[PLU007C94] Keeling MJ, Danon L, Vernon MC, House TA (2010). Individual identity and movement networks for disease metapopulations. Proceedings of the National Academy of Sciences of the USA.

[PLU007C95] Klemm K, Serrano MA, Eguíluz VM, San Miguel M (2012). A measure of individual role in collective dynamics. Scientific Reports.

[PLU007C96] Kretzschmar M (2000). Sexual network structure and sexually transmitted disease prevention: a modeling perspective. Sexually Transmitted Diseases.

[PLU007C97] Kueffer C, Pyšek P, Richardson DM (2013). Integrative invasion science: model systems, multi-site studies, focused meta-analysis and invasion syndromes. New Phytologist.

[PLU007C98] Latty T, Ramsch K, Ito K, Nakagaki T, Sumpter DJT, Middendorf M, Beekman M (2011). Structure and formation of ant transportation networks. Interface.

[PLU007C99] Leventhal GE, Kouyos R, Stadler T, Von Wyl V, Yerly S, Böni J, Cellerai C, Klimkait T, Günthard HF, Bonhoeffer S (2012). Inferring epidemic contact structure from phylogenetic trees. PLoS Computational Biology.

[PLU007C100] Liebhold AM, Brockerhoff EG, Garrett LJ, Parke JL, Britton KO (2012). Live plant imports: the major pathway for forest insect and pathogen invasions of the US. Frontiers in Ecology and the Environment.

[PLU007C101] Maciejewski R, Livengood P, Rudolph S, Collins TF, Ebert DS, Brigantic RT, Corley CD, Muller GA, Sanders SW (2011). A pandemic influenza modeling and visualization tool. Journal of Visual Languages & Computing.

[PLU007C102] MacLeod A, Pautasso M, Jeger MJ, Haines-Young R (2010). Evolution of the international regulation of plant pests and challenges for future plant health. Food Security.

[PLU007C103] Mascheretti S, Croucher PJP, Kozanitas M, Baker L, Garbelotto M (2009). Genetic epidemiology of the Sudden Oak Death pathogen *Phytophthora ramorum* in California. Molecular Ecology.

[PLU007C104] May RM (2006). Network structure and the biology of populations. Trends in Ecology and Evolution.

[PLU007C105] May RM (2007). Parasites, people and policy: infectious diseases and the Millennium Development Goals. Trends in Ecology and Evolution.

[PLU007C106] McMichael AJ, Beaglehole R (2000). The changing global context of public health. The Lancet.

[PLU007C107] Merler S, Ajelli M (2010a). Human mobility and population heterogeneity in the spread of an epidemic. Procedia Computer Science.

[PLU007C108] Merler S, Ajelli M (2010b). The role of population heterogeneity and human mobility in the spread of pandemic influenza. Proceedings of the Royal Society B Biological Sciences.

[PLU007C109] Merler S, Ajelli M, Pugliese A, Ferguson NM (2011). Determinants of the spatiotemporal dynamics of the 2009 H1N1 pandemic in Europe: implications for real-time modelling. PLoS Computational Biology.

[PLU007C110] Meyers LA, Newman MEJ, Pourbohloul B (2006). Predicting epidemics on directed contact networks. Journal of Theoretical Biology.

[PLU007C111] Miller JC, Slim AC, Volz EM (2012). Edge-based compartmental modelling for infectious disease spread. Interface.

[PLU007C112] Miller SA, Beed FD, Harmon CL (2009). Plant disease diagnostic capabilities and networks. Annual Review of Phytopathology.

[PLU007C113] Morris M, Kretzschmar M (1995). Concurrent partnerships and transmission dynamics in networks. Social Networks.

[PLU007C114] Moslonka-Lefebvre M, Pautasso M, Jeger MJ (2009). Disease spread in small-size directed networks: epidemic threshold, correlation between links to and from nodes, and clustering. Journal of Theoretical Biology.

[PLU007C115] Moslonka-Lefebvre M, Finley A, Dorigatti I, Dehnen-Schmutz K, Harwood T, Jeger MJ, Xu XM, Holdenrieder O, Pautasso M (2011). Networks in plant epidemiology: from genes to landscapes, countries and continents. Phytopathology.

[PLU007C116] Moslonka-Lefebvre M, Harwood T, Jeger MJ, Pautasso M (2012). SIS along a continuum (SISc) epidemiological modelling and control of diseases on directed trade networks. Mathematical Biosciences.

[PLU007C117] Mossong J, Hens N, Jit M, Beutels P, Auranen K, Mikoljczyk R, Massari M, Salmaso S, Scalia Tomba G, Wallinga J, Heijne J, Sadkowska-Todys M, Rosinska M, Edmunds JW (2008). Social contacts and mixing patterns relevant to the spread of infectious diseases. PLoS Medicine.

[PLU007C118] Natale F, Giovannini A, Savini L, Palma D, Possenti L, Fiore G, Calistri P (2009). Network analysis of Italian cattle trade patterns and evaluation of risks for potential disease spread. Preventive Veterinary Medicine.

[PLU007C119] Natale F, Savini L, Giovannini A, Calistri P, Candeloro L, Fiore G (2011). Evaluation of risk and vulnerability using a disease flow centrality measure in dynamic cattle trade networks. Preventive Veterinary Medicine.

[PLU007C120] Newman MEJ (2002). Spread of epidemic disease on networks. Physical Review E.

[PLU007C121] Newman MEJ (2003). The structure and function of complex networks. SIAM Review.

[PLU007C122] Newman MEJ (2006). Modularity and community structure in networks. Proceedings of the National Academy of Sciences of the USA.

[PLU007C123] Newman MEJ, Park J (2003). Why social networks are different from other types of networks. Physical Review E.

[PLU007C124] Newman MEJ, Strogatz SH, Watts DJ (2001). Random graphs with arbitrary degree distributions and their applications. Physical Review E.

[PLU007C125] Nickbakhsh S, Matthews L, Bessell PR, Reid SWJ, Kao RR (2011). Generating social network data using partially described networks: an example informing avian influenza control in the British poultry industry. BMC Veterinary Research.

[PLU007C126] Nöremark M, Hakansson N, Sternberg Lewerin S, Lindberg A, Jonsson A (2011). Network analysis of cattle and pig movements in Sweden: measures relevant for disease control and risk based surveillance. Preventive Veterinary Medicine.

[PLU007C127] Ochab JK, Gora PF (2011). Shift of percolation thresholds for epidemic spread between static and dynamic small-world networks. European Physical Journal B.

[PLU007C128] Oliveira M, Gama J (2012). An overview of social network analysis. Wiley Interdisciplinary Reviews: Data Mining and Knowledge Discovery.

[PLU007C129] Pan RK, Saramäki J (2011). Path lengths, correlations, and centrality in temporal networks. Physical Review E.

[PLU007C130] Pastor-Satorras R, Vespignani A (2001). Epidemic spreading in scale-free networks. Physical Review Letters.

[PLU007C131] Pautasso M (2013). *Phytophthora ramorum*—a pathogen linking network epidemiology, landscape pathology and conservation biogeography. CAB Reviews.

[PLU007C132] Pautasso M, Dehnen-Schmutz K, Holdenrieder O, Pietravalle S, Salama N, Jeger MJ, Lange E, Hehl-Lange S (2010a). Plant health and global change—some implications for landscape management. Biological Reviews.

[PLU007C133] Pautasso M, Moslonka-Lefebvre M, Jeger MJ (2010b). The number of links to and from the starting node as a predictor of epidemic size in small-size directed networks. Ecological Complexity.

[PLU007C134] Pautasso M, Xu X-M, Jeger MJ, Harwood TD, Moslonka-Lefebvre M, Pellis L (2010c). Disease spread in small-size directed trade networks: the role of hierarchical categories. Journal of Applied Ecology.

[PLU007C135] Pautasso M, Dehnen-Schmutz K, Ilbery B, Jeger MJ, Jones G, Little R, MacLeod A, Maye D, Parker S, Pietravalle S, Mills P (2012). Plant health challenges for a sustainable land use and rural economy. CAB Reviews.

[PLU007C136] Perisic A, Bauch CT (2009). Social contact networks and disease eradicability under voluntary vaccination. PLoS Computational Biology.

[PLU007C137] Pongsiri MJ, Roman J, Ezenwa VO, Goldberg TL, Koren HS, Newbold SC, Ostfeld RS, Pattanayak SK, Salkeld DJ (2009). Biodiversity loss affects global disease ecology. BioScience.

[PLU007C138] Potter GE, Handcock MS, Longini IM, Halloran ME (2011). Estimating within-household contact networks from egocentric data. Annals of Applied Statistics.

[PLU007C139] Prieto DM, Das TK, Savachkin AA, Uribe A, Izurieta R, Malavade S (2012). A systematic review to identify areas of enhancements of pandemic simulation models for operational use at provincial and local levels. BMC Public Health.

[PLU007C140] Queloz V, Grünig CR, Berndt R, Kowalski T, Sieber TN, Holdenrieder O (2011). Cryptic speciation in *Hymenoscyphus albidus*. Forest Pathology.

[PLU007C141] Rabinowitz HK, Diamond JJ, Markham FW, Hazelwood CE (1999). A program to increase the number of family physicians in rural and underserved areas. Impact after 22 years. JAMA.

[PLU007C142] Rebaudo F, Dangles O (2011). Coupled information diffusion–pest dynamics models predict delayed benefits of farmer cooperation in pest management programs. PLoS Computational Biology.

[PLU007C143] Redlin SC, Werres S, Schröder T, Gordh G, McKirdy S (2014). Invasive pathogens in plant biosecurity. Case study: *Phytophthora ramorum* Werres *et al.*: cause of Sudden Oak Death, ramorum leaf blight and ramorum dieback. The handbook of plant biosecurity.

[PLU007C144] Reppas AI, Spiliotis KG, Siettos CI (2010). Epidemionics. From the host–host interactions to the systematic analysis of the emergent macroscopic dynamics of epidemic networks. Virulence.

[PLU007C145] Ridenhour BJ, Braun A, Teyrasse T, Goldsman D (2011). Controlling the spread of disease in schools. PLoS One.

[PLU007C146] Robinson K, Cohen T, Colijn C (2012). The dynamics of sexual contact networks: effects on disease spread and control. Theoretical Population Biology.

[PLU007C147] Rock K, Brand S, Moir J, Keeling MJ (2014). Dynamics of infectious diseases. Reports on Progress in Physics.

[PLU007C148] Rohani P, Zhong X, King AA (2010). Contact network structure explains the changing epidemiology of pertussis. Science.

[PLU007C149] Rothenberg R, Narramore J (1996). The relevance of social network concepts to sexually transmitted disease control. Sexually Transmitted Diseases.

[PLU007C150] Rozhnova G, Nunes A, McKane AJ (2011). Stochastic oscillations in models of epidemics on a network of cities. Physical Review E.

[PLU007C151] Ruzzenenti F, Garlaschelli D, Basosi R (2010). Complex networks and symmetry II: reciprocity and evolution of world trade. Symmetry.

[PLU007C152] Saadatian-Elahi M, Facy F, Del Signore C, Vanhems P (2010). Perception of epidemic's related anxiety in the general French population: a cross-sectional study in the Rhône-Alpes region. BMC Public Health.

[PLU007C153] Salathé M, Kazandjieva M, Lee JW, Levis P, Feldman MW, Jones JH (2010). A high-resolution human contact network for infectious disease transmission. Proceedings of the National Academy of Sciences of the USA.

[PLU007C154] Schoebel CN, Stewart J, Gruenwald NJ, Rigling D, Prospero S (2014). Population history and pathways of spread of the plant pathogen *Phytophthora plurivora*. PLoS One.

[PLU007C155] Schweitzer F, Fagiolo G, Sornette D, Vega-Redondo F, Vespignani A, White DR (2009). Economic networks: the new challenges. Science.

[PLU007C156] Shirey PD, Kunycky BN, Chaloner DT, Brueseke MA, Lamberti GA (2013). Commercial trade of federally listed threatened and endangered plants in the United States. Conservation Letters.

[PLU007C157] Shirley MDF, Rushton SP (2005). The impacts of network topology on disease spread. Ecological Complexity.

[PLU007C158] Smieszek T (2010). Models of epidemics: how contact characteristics shape the spread of infectious diseases. http://e-collection.library.ethz.ch/eserv/eth:1519/eth-1519-02.pdf.

[PLU007C159] Smilkov D, Kocarev L (2012). Influence of the network topology on epidemic spreading. Physical Review E.

[PLU007C160] Smith KP, Christakis NA (2008). Social networks and health. Annual Review of Sociology.

[PLU007C161] Smith VS (2007). Clean: a history of personal hygiene and purity.

[PLU007C162] Song C, Qu Z, Blumm N, Barabási AL (2010a). Limits of predictability in human mobility. Science.

[PLU007C163] Song C, Koren T, Wang P, Barabási AL (2010b). Modelling the scaling properties of human mobility. Nature Physics.

[PLU007C164] Stehlé J, Barrat A, Bianconi G (2010). Dynamical and bursty interactions in social networks. Physical Review E.

[PLU007C165] Stehlé J, Voirin N, Barrat A, Cattuto C, Isella L, Pinton J-F, Quaggiotto M, Van Den Broeck W, Regis C, Lina B, Vanhems P (2011). High-resolution measurements of face-to-face contact patterns in a primary school. PLoS One.

[PLU007C166] Stone TE, McKay SR (2011). Critical behavior of disease spread on dynamic small-world networks. EPL.

[PLU007C167] Stone TE, Jones MM, Mckay SR (2010). Comparative effects of avoidance and vaccination in disease spread on a dynamic small-world network. Physica A.

[PLU007C168] Stumpf MPH, Wiuf C, May RM (2005). Subnets of scale-free networks are not scale-free: sampling properties of networks. Proceedings of the National Academy of Sciences of theUSA.

[PLU007C169] Sutrave S, Scoglio C, Isard SA, Hutchinson JMS, Garrett KA (2012). Identifying highly connected counties compensates for resource limitations when evaluating national spread of an invasive pathogen. PLoS One.

[PLU007C170] van der Graaff NA, Khoury W, Strange RN, Gullino ML (2010). Biosecurity in the movement of commodities as a component of global food security. The role of plant pathology in food safety and food security.

[PLU007C171] Vernon MC, Keeling MJ (2009). Representing the UK's cattle herd as static and dynamic networks. Proceedings of the Royal Society B Biological Sciences.

[PLU007C172] Vespignani A (2012). Modelling dynamical processes in complex socio-technical systems. Nature Physics.

[PLU007C173] Viboud C, Bjornstad ON, Smith DL, Simonsen L, Miller MA, Grenfell BT (2006). Synchrony, waves, and spatial hierarchies in the spread of influenza. Science.

[PLU007C174] Volz E, Meyers LA (2009). Epidemic threshold in dynamic contact networks. Interface.

[PLU007C175] Wang S, Noe RA (2010). Knowledge sharing: a review and directions for future research. Human Resource Management Review.

[PLU007C176] Watts DJ, Strogatz SH (1998). Collective dynamics of ‘small-world’ networks. Nature.

[PLU007C177] Weiss RA, McMichael AJ (2004). Social and environmental risk factors in the emergence of infectious diseases. Nature Medicine.

[PLU007C178] Welch D, Bansal S, Hunter DR (2011). Statistical inference to advance network models in epidemiology. Epidemics.

[PLU007C179] Weng L, Flammini A, Vespignani A, Menczer F (2012). Competition among memes in a world with limited attention. Scientific Reports.

[PLU007C180] Woolhouse M, Gaunt E (2007). Ecological origins of novel human pathogens. Critical Reviews in Microbiology.

[PLU007C181] Woolhouse MEJ, Shaw DJ, Matthews L, Liu WC, Mellor DJ, Thomas MR (2005). Epidemiological implications of the contact network structure for cattle farms and the 20–80 rule. Biology Letters.

[PLU007C182] Woolley-Meza O, Thiemann C, Grady D, Lee JJ, Seebens H, Blasius B, Brockmann D (2011). Complexity in human transportation networks: a comparative analysis of worldwide air transportation and global cargo-ship movements. European Physical Journal B.

[PLU007C183] Xhaard C, Barres B, Andrieux A, Bousset L, Halkett F, Frey P (2012). Disentangling the genetic origins of a plant pathogen during disease spread using an original molecular epidemiology approach. Molecular Ecology.

[PLU007C184] Xu X, Harwood TD, Pautasso M, Jeger MJ (2009). Spatio-temporal analysis of an invasive plant pathogen (*Phytophthora ramorum*) in England and Wales. Ecography.

[PLU007C185] Yemshanov D, Koch FH, Ducey MJ, Siltanen M, Wilson K, Koehler K (2013). Exploring critical uncertainties in pathway assessments of human-assisted introductions of alien forest species in Canada. Journal of Environmental Management.

[PLU007C186] Ypma RJF, Bataille AMA, Stegeman A, Koch G, Wallinga J, Van Ballegooijen WM (2012). Unravelling transmission trees of infectious diseases by combining genetic and epidemiological data. Proceedings of the Royal Society London B Biological Sciences.

[PLU007C187] Ypma RJF, Van Ballegooijen WM, Wallinga J (2013). Relating phylogenetic trees to transmission trees of infectious disease outbreaks. Genetics.

[PLU007C188] Zappa P (2011). The network structure of knowledge sharing among physicians. Quality & Quantity.

[PLU007C189] Zhao Y, Levina E, Zhu J (2011). Community extraction for social networks. Proceedings of the National Academy of Sciences of the USA.

